# mLoc-mRNA: predicting multiple sub-cellular localization of mRNAs using random forest algorithm coupled with feature selection via elastic net

**DOI:** 10.1186/s12859-021-04264-8

**Published:** 2021-06-24

**Authors:** Prabina Kumar Meher, Anil Rai, Atmakuri Ramakrishna Rao

**Affiliations:** ICAR-Indian Agricultural Statistics Research Institute, New Delhi, 110012 India

**Keywords:** Sub-cellular localization, Machine learning, Feature selection, Computational biology, Bioinformatics

## Abstract

**Background:**

Localization of messenger RNAs (mRNAs) plays a crucial role in the growth and development of cells. Particularly, it plays a major role in regulating spatio-temporal gene expression. The in situ hybridization is a promising experimental technique used to determine the localization of mRNAs but it is costly and laborious. It is also a known fact that a single mRNA can be present in more than one location, whereas the existing computational tools are capable of predicting only a single location for such mRNAs. Thus, the development of high-end computational tool is required for reliable and timely prediction of multiple subcellular locations of mRNAs. Hence, we develop the present computational model to predict the multiple localizations of mRNAs.

**Results:**

The mRNA sequences from 9 different localizations were considered. Each sequence was first transformed to a numeric feature vector of size 5460, based on the *k*-mer features of sizes 1–6. Out of 5460 *k*-mer features, 1812 important features were selected by the Elastic Net statistical model. The Random Forest supervised learning algorithm was then employed for predicting the localizations with the selected features. Five-fold cross-validation accuracies of 70.87, 68.32, 68.36, 68.79, 96.46, 73.44, 70.94, 97.42 and 71.77% were obtained for the cytoplasm, cytosol, endoplasmic reticulum, exosome, mitochondrion, nucleus, pseudopodium, posterior and ribosome respectively. With an independent test set, accuracies of 65.33, 73.37, 75.86, 72.99, 94.26, 70.91, 65.53, 93.60 and 73.45% were obtained for the respective localizations. The developed approach also achieved higher accuracies than the existing localization prediction tools.

**Conclusions:**

This study presents a novel computational tool for predicting the multiple localization of mRNAs. Based on the proposed approach, an online prediction server “mLoc-mRNA” is accessible at http://cabgrid.res.in:8080/mlocmrna/. The developed approach is believed to supplement the existing tools and techniques for the localization prediction of mRNAs.

**Supplementary Information:**

The online version contains supplementary material available at 10.1186/s12859-021-04264-8.

## Background

The discovery of asymmetrical distribution of $\beta$-actin mRNA in ascidian embryos and eggs by Jeffery et al. [1] laid the foundation for mRNA localization study. However, valuable understandings of mRNA localization are stemmed from the research undertaken thereafter in fungi and animals [2–6]. Localization of mRNAs plays a prominent role in spatio-temporal regulation of gene expression, which is crucial for different cellular and developmental processes including asymptotic cell division, cell migration, embryonic patterning and cellular adaptation to stress [2, 3, 7, 8]. Localization of mRNAs further facilitates sub-cellular localization of proteins that helps to establish and maintain the cell polarity [2]. In the nervous system of mammals, localization of mRNAs plays vital roles ranging from axon and dendrite path finding to synapse generation [9, 10]. Maintenance of synaptic plasticity responsible for long-lasting learning and memory also depends upon the localization of mRNAs [11–13]. Deregulation of mRNA stability and localization may cause different genetic disorders including cancer [9]. More details on the adverse impact of the deregulation of mRNA localizations in animals can be found in existing studies [14–16].

A crucial factor for the localization of mRNAs is the recognition of cis-acting signals (or zipcodes) by the trans-acting factors (mostly RNA binding proteins)[17]. The cis-acting elements are mostly present in 3’UTR of mRNAs [3, 18–20]. Localization of mRNAs takes place through three mechanisms i.e., vectorial transport from nuclei, localized protection from degradation and directional transport on the cytoskeleton via molecular motors [2, 3]. Since a single localized mRNA can produce multiple protein copies, the localization of mRNAs saves energy for a cell [21]. Besides, the locally translated proteins prevent themselves from interaction with the other proteins as well as from synthesis in the incorrect locations that is harmful to the cell [22]. The mRNA localization also facilitates the assembly of macromolecular protein complexes [23] and regulates the differential translation as well [24, 25].

Although in situ hybridization is a reliable experimental technique for mRNA localization, it is a slow and laborious approach. The in situ hybridization allows the rapid labeling of mRNAs [26, 27] but it is limited to certain tissues [28]. With the advancement of in vivo mRNA localization techniques such as MS2-PP7 [29] system, total internal reflection microscopy (TIRF) [30] and 3D structured illumination microscopy (3D-SIM) [31], a large number of localized mRNAs are available in the public domain. Hence, the development of computational tools for mRNA localization prediction has now become feasible.

Predicting the localization of mRNAs can be formulated as both supervised and unsupervised learning problems. In the case of unsupervised learning, homology-based methods such as BLAST [32] and HMM [33] can be used for predicting the localization of mRNAs. By using BLAST, a database of all the localized mRNA sequences can be built and based on the blast search each query sequence can be assigned to a particular localization depending upon the sequence similarity found. Similarly, an HMM profile can be created by using all the localization datasets and the localization of the query sequence can be searched against the created HMM profile. However, by using such models only a single localization can be predicted for any mRNA. On the other hand, if different databases/profiles are created for different localizations to predict multiple localizations, probability of getting a large number of false positives is always there. This may be the reason the existing tools are based on the supervised learning model. The supervised computational tools such as RNATracker [34], iLoc-mRNA [35], and mRNALoc [36] have been developed for predicting the mRNA localization. It is a well-known fact that a single mRNA could be present in more than one localization, whereas the existing tools are meant for predicting single localization only. In other words, only a single localization can be predicted for each mRNA by using the existing tools. The use of CeFra-Seq [37] and APEX-RIP [38] datasets in RNATracker further limits its application. Also, the numbers of localizations considered in the existing tools are not more than five.

The above-stated factors prompted us to develop a new computational model for predicting the multiple localization of mRNAs. The *k*-mer features were generated to translate the mRNA sequences into vectors of numeric elements. The Elastic Net [39] statistical model was used for the selection of important *k*-mer features. The Random Forest [40] supervised learning model was employed for predicting the localizations using the selected *k*-mer features. The performance of the model was evaluated by following the cross-validation and also by using the independent datasets. The proposed model achieved higher accuracy in most of the localizations, while compared using the independent test datasets. We have also developed an online prediction server for the multiple subcellular localization prediction of mRNAs. The developed model is expected to supplement the existing localization prediction tools in particular, and the wet-lab experiments in general.

## Methods

### Collection and processing of localization dataset

**Fig. 1:**
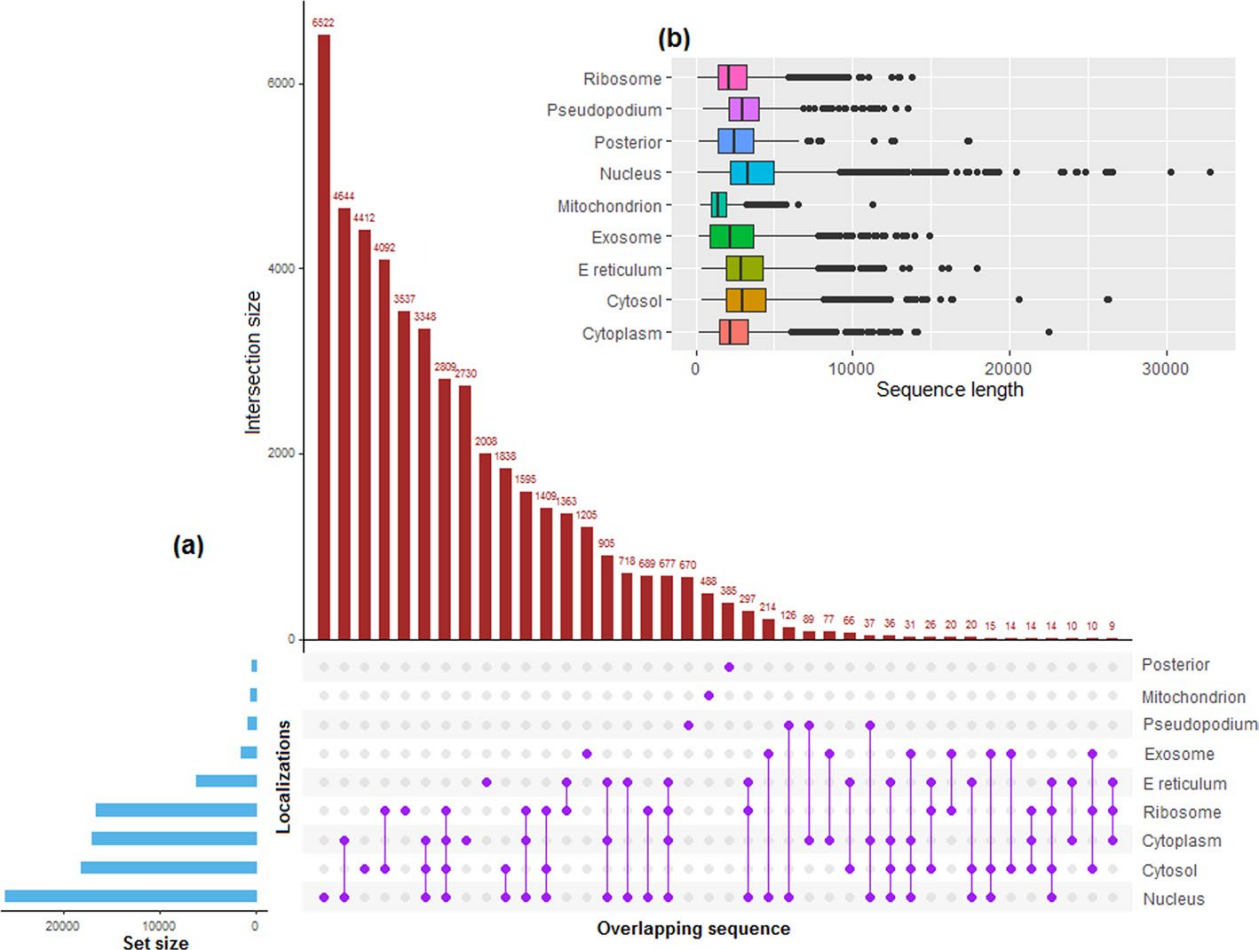
**a** Pictorial representation of the number of overlapping mRNA sequences in different localizations. The purple dots represent the number of overlapping localizations. For instance, between nucleus and cytoplasm there are 4644 overlapped sequences. Except mitochondrion and posterior, mRNA sequences of the remaining localizations are seen to be overlapped. **b** Box plot showing the distribution of sequence lengths for different localizations. It can be noticed that the variability in the sequence lengths are different for different localizations, where the highest variability is for the nucleus and lowest for the mitochondrion

The mRNA sequences of 9 localizations i.e., cytoplasm (17,053), cytosol (18,173), endoplasmic reticulum (6179), exosome (1623), mitochondrion (525), nucleus (25,981), pseudopodium (923), posterior (402) and ribosome (16,602) were collected from the RNALocate database [41] (order of the locations will remain same hereafter). Except for the posterior and mitochondrion, sequences of the remaining localizations were found to be overlapping (Fig. 1a). In other words, the sequences were seen to be present in more than one localization and up to a maximum of 4 localizations. For instance, 2809 sequences were found common in the cytoplasm, cytosol, nucleus and ribosome (represented as purple dots in Fig. 1a). After removing the overlapped sequences, 2730, 4412, 2008, 1205, 488, 6522, 670, 385 and 3537 mRNA sequences were obtained for the respective localizations. Since a higher degree of sequence similarity may lead to overestimation of the prediction accuracy, CD-HIT [42] was employed with an 80% cut-off to remove the redundant sequences from each localization. The 80% threshold has also been used in earlier studies [35, 43]. After removing the redundancy, 1804, 2158, 1020, 843, 457, 3304, 216, 187 and 1838 sequences were obtained for the corresponding localizations. Variability in the sequence length was also observed to be different for different localizations, with the highest variability for the nucleus and lowest for the mitochondrion (Fig. 1b).

### Preparation of the positive, negative and independent data sets

**Fig. 2:**
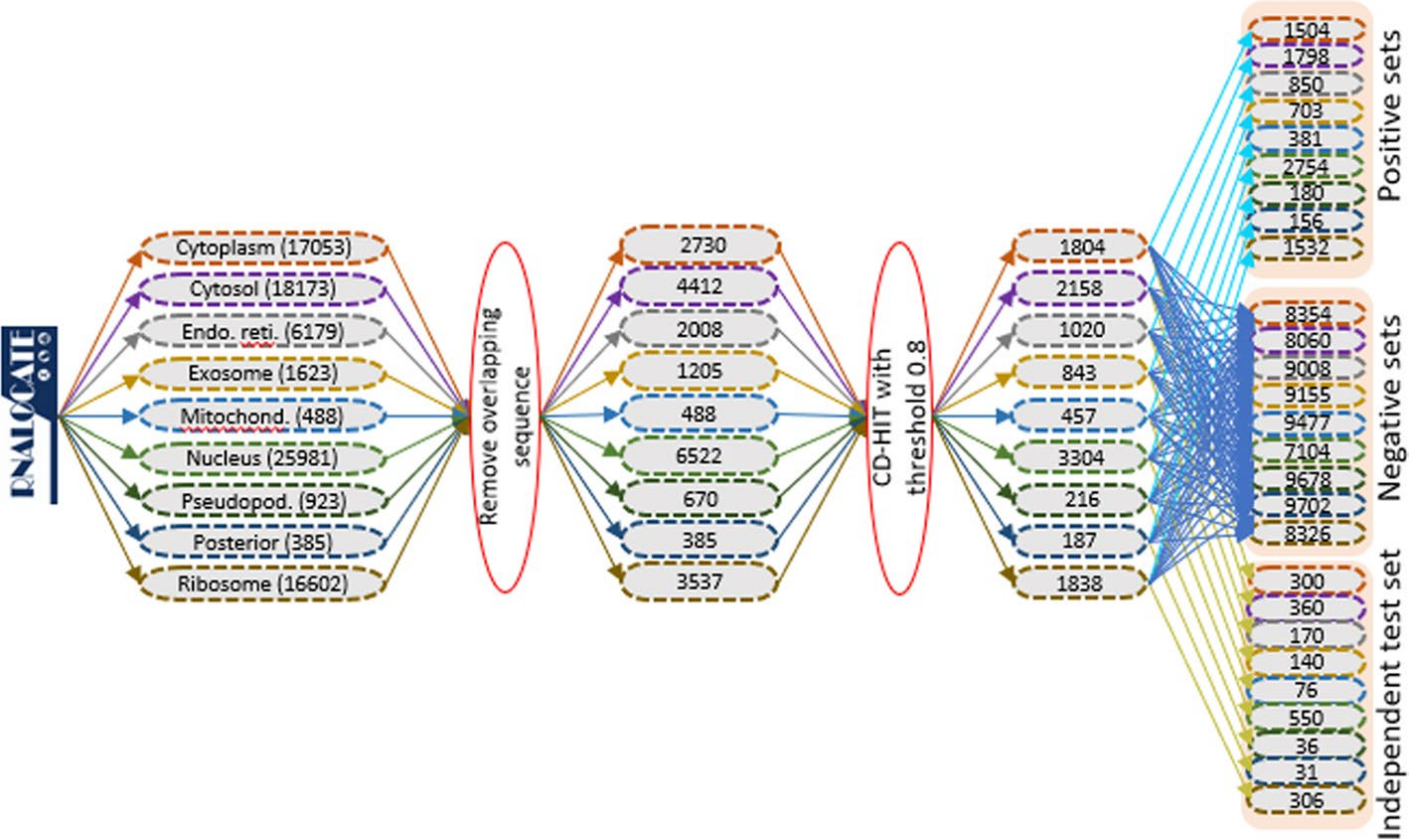
Diagramatic representation of the collection of localization datatset as well as preparation of the positive, negative and independent datasets

A single mRNA can be present in multiple subcellular locations. So, by using a single multi-class prediction model trained with 9 different classes (corresponding to 9 localizations), only a single localization (localization with the highest probability) can be predicted for each mRNA sequence. Therefore, it is required to establish multiple binary classifiers instead of a single multi-class classifier to predict the multiple localizations of mRNAs. Specifically, 9 binary classifiers are required for 9 localizations. Bu using 9 binary classifiers, the probability of each mRNA predicted in nine different localizations can be obtained. Then, with the probability threshold value 0.5, the localization (one or more) can be easily decided for the given mRNA sequence. Keeping above in mind, positive and negative datasets were prepared for each of the 9 binary classifiers. The sequence datasets obtained after removing the redundancy were randomly divided into 6 equal-sized subsets for each localization, where one randomly drawn subset from each localization was kept aside to use them as an independent dataset. The remaining 5 subsets of each localization were used to train and validate the classifiers, keeping in mind the five-fold cross-validation. In particular, 300, 360, 170, 140, 76, 550, 36, 31, 306 sequences were used as an independent set (Independent test set-I) and the remaining 1504, 1798, 850, 703, 381, 2754, 180, 156, 1532 sequences of the respective localizations were used for training and validating the classifiers (Fig. 2) through five-fold cross validation. Further, the number of training sequences for the pseudopodium (180) and posterior (156) were not large. Thus, if more sequences would be considered in the test set then the number of sequences for the training would be less. Similarly, the numbers of test sequences for these two localizations (36, 31) were also less. So, if more sequences would be taken in the training set, then a very few sequences would be left out for the test set. Thus, we did not consider any other combination of the training and test sets. Nevertheless, more stable accuracy can be obtained by training the model with a large dataset. For a given localization, sequences of that localization were used as the positive set and the sequences of the remaining 8 localizations were utilized as the negative set. For instance, 1504 sequences were used as the positive set for the cytoplasm and 8354 (1798 + 850 + 703 + 381 + 2754 + 180 + 156 + 1532) sequences of the remaining localizations were utilized as the negative set. Similarly, the positive and negative datasets were also prepared for other localizations. Besides Independent test set-I, we prepared another independent dataset (i.e., Independent test set-II) with the CD-HIT filtered-out sequences after removing redundancy at 80% threshold. In the Independent test set-II, there were 490, 1037, 485, 185, 14, 1266, 79, 121, 798 sequences for the respective localizations. A graphical representation with the steps involved for preparing the positive, negative and independent datasets is shown in Fig. 2. A summary of the number of positive and negative datasets used in training and test sets are provided in Additional file 1: Table S1.

### *K*-mer feature generation

The *k*-mer features have been effectively and widely used in many bioinformatics applications including sequence alignment, genome assembly, characterization of microbial community and others [44–46]. These features have also been widely adopted for the recognition of regulatory elements on the genomic DNA/RNA [47–49]. The *k*-mer is a fragment of sequence containing *k* oligonucleotides, where the total number of possible *k*-mer is 4^ k^. We employed *k-*mer sizes 1, 2, 3, 4, 5, 6, and hence the total number of features generated was 5460 (4^1^ + 4^2^ + 4^3^ + 4^4^ + 4^5^ + 4^6^). Besides the “curse of dimensionality”, using such a large number of features may result in low prediction accuracy due to the presence of many irrelevant/redundant features [48, 50–52]. Over fitting of the model may be another issue with a large number of features, which results in low generalization predictive ability of the model [43]. These limitations could be overcome to a large extent by applying the feature selection technique.

### Feature selection using elastic net

The Elastic Net [39] statistical model, which is a combination of the LASSO [53] and Ridge regression [54] algorithms, was employed for the selection of important *k*-mer features. Consider the generalized linear model

$$\boldsymbol{Y}=\boldsymbol{X}\beta +\epsilon ,$$

where ${\boldsymbol{Y}}_{\boldsymbol{n}\times 1}$ is the response for *n* observations, ${\boldsymbol{X}}_{\boldsymbol{n}\times \boldsymbol{p}}$ is the design matrix for *p* variables, $\beta$ is the vector of coefficients and $\epsilon$ is the vector of random errors. For this model, the estimates using the Elastic Net method can be obtained as

$$\hat{\beta }=arg\underset{\beta }{min}\left(\frac{||\boldsymbol{Y}-\boldsymbol{X}\beta |{|}_{2}^{2}}{n}+\lambda \left(1-\alpha \right)\left|\left|\beta \right|{|}^{2}+\lambda \alpha \right|\left|\beta \right|{|}_{1}\right).$$

The parameter $\lambda (\ge 0)$ controls the amount of shrinkage, where $\lambda$=0 represents an ordinary least square solution, and all the coefficients shrink to zero with $\lambda =\infty$. The regularization parameter $\alpha$ controls both the Ridge and LASSO, where $\alpha =0$ and 1 deduce the Ridge and LASSO models respectively. In Elastic Net, the coefficients of the least important variables are shrunk to zero and the variables with the non-zero coefficients are considered important. We implemented the Elastic Net using “glmnet” R-package [55], where the classification task was performed with binary response variable by invoking the binomial distribution with parameter *family* = *“binomial”*. By using repeated five-fold cross-validation approach, the optimum value of $\alpha$ (based on highest classification accuracy) was selected out of 10 different values of $\alpha$ i.e., 0.1, 0.2, 0.3, 0.4, 0.5, 0.6, 0.7, 0.8, 0.9 and 1. Using the optimum value of $\alpha$, the function *cv.glmnet* (with *nfolds* = *5*) was then employed to find the optimum value of $\lambda$ (with respect to classification accuracy) through a grid search approach. Parameters were optimized with 50% random sample observations of the training set, having same number of instances from both the positive and negative classes.

### Prediction using random forest

**Fig. 3:**
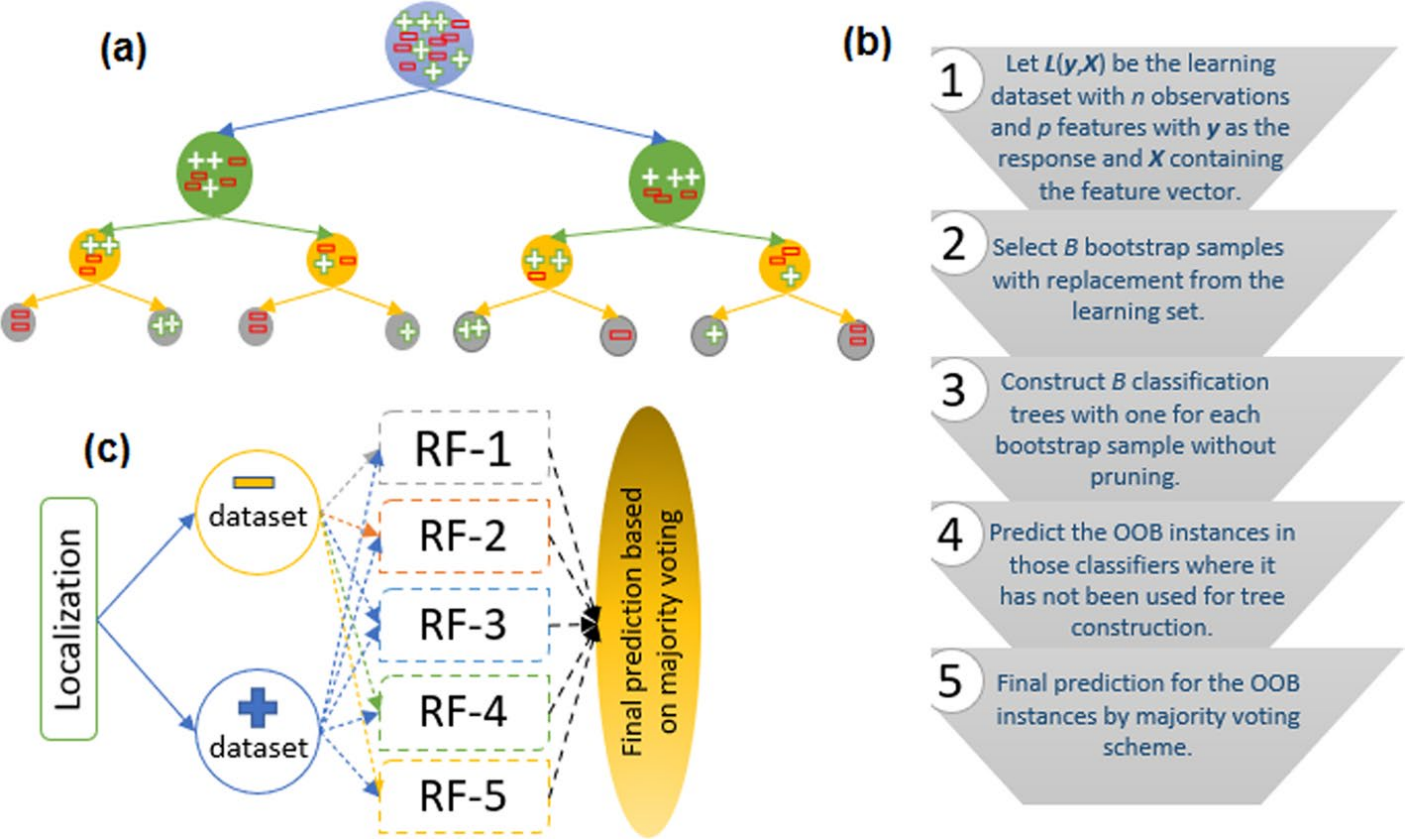
**a** Pictorial representation of a classification tree. **b** Diagram showing the steps for building a Random Forest (RF) classifier. **c** Graphical display of the prediction using RF for each localization. For each localization, 5 RF classifiers are built and the final prediction results are determined on the basis of majority voting scheme. Further, each RF classifier is trained with a balanced dataset that consists of all the positive instances and same number of randomly drawn negative instances. The negative dataset is different for all the five RF classifiers for every localizations

**Fig. 4:**
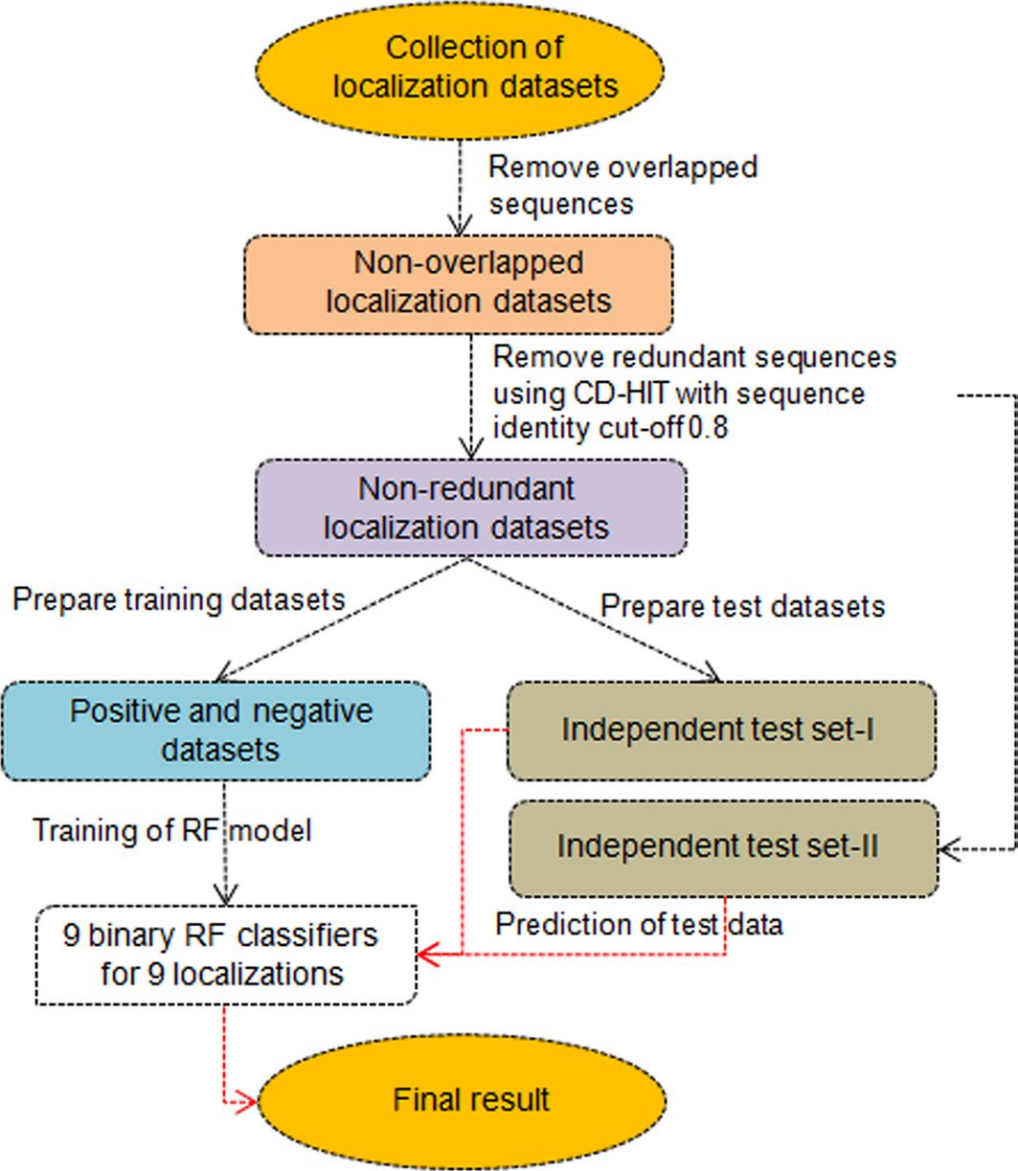
A flow diagram of the proposed method comprising the steps from collection of data to prediction of test instances

We employed the Random Forest (RF) [40] supervised learning algorithm for binary classification. The RF is an ensemble of several un-pruned classification trees (Fig. 3a), where each classification tree is grown upon a bootstrap sample of the training dataset [56, 57]. The decision tree starts with the root node containing all the observations and ends with the terminal nodes having the labels of observations (Fig. 3a). In RF, randomization is introduced at two stages, (1) while drawing the bootstrap sample and, (2) during the splitting of the nodes of the classification tree. Each classification tree of RF votes for every such instance that does not participate in the construction of the classifier and such instances are called out-of-bag (OOB) instances. The OOB observations are the source to estimate the classification error in RF [58], where the prediction label of each instance is decided by the majority voting scheme. The steps for building an RF classifier are shown in Fig. 3b. The RF is a non-parametric technique, handles large size datasets, provides higher accuracy even for noisy data and overcomes the problem of over-fitting with bootstrapping and ensemble strategy [59]. The *ntree* (number of classification trees) and *mtry* (number of variables to be selected for node splitting) are the parameters duo that are needed to be optimized to achieve higher accuracy. Since default parametric values often produce higher accuracy [60], we used *ntree* = 500 (default *ntree*) and default *mtry* i.e., square root of the number of features. Further, a larger number of instances for the negative class as compared to its positive counterpart (Fig. 2) may lead to the prediction bias towards the negative class. To overcome this problem, we employed 5 RF classifiers (instead of one) for each localization. In each RF classifier, all the instances of the positive class and an equal number of instances randomly drawn from the negative class were utilized. A five-fold cross-validation approach was adopted to measure the accuracy for each RF and a majority voting strategy was applied for the final prediction (Fig. 3c). In other words, if an instance was predicted to a certain class in 3 out of 5 RF classifiers the instance was said to be predicted in that class. This procedure was followed for all the 9 binary classifiers. A flow diagram showing the steps involved in the proposed approach is shown in Fig. 4.

### Performance metrics

Five different performance metrics were used to measure the classification accuracy. The metrics are

- sensitivity =  $$\frac{{N}^{+}-{N}_{-}^{+}}{{N}^{+}}$$,
- specificity =  $$\frac{{N}^{-}-{N}_{+}^{-}}{{N}^{-}}$$,
- accuracy =  $$\frac{{N}^{-}-{N}_{+}^{-}+{N}^{+}-{N}_{-}^{+}}{{N}^{-}+{N}^{+}}$$,
- Matthew’s correlation coefficient (MCC) = $\frac{1-\left(\frac{{N}_{-}^{+}}{{N}^{+}}+\frac{{N}_{+}^{-}}{{N}^{-}}\right)}{\sqrt{\left(1+\frac{{N}_{-}^{+}-{N}_{+}^{-}}{{N}^{+}}\right)\left(1+\frac{{N}_{+}^{-}-{N}_{-}^{+}}{{N}^{-}}\right)}}$ and
- F1-score = $$\frac{{N}^{+}-{N}_{-}^{+}}{\left({N}^{+}-{N}_{-}^{+}\right)+\frac{1}{2}\left({N}_{+}^{-}+{N}_{-}^{+}\right)}$$,

where ${N}^{+}$, ${N}^{-}$, ${N}_{-}^{+}$ and ${N}_{+}^{-}$ represent the number of observations of the positive class, number of observations of the negative class, number of positive observations misclassified in the negative class and the number of negative observations misclassified in the positive class respectively [61–64]. We also computed area under receiver-operating-characteristics (ROC) [65] curve (aucROC) and area under precision-recall (PR) curve (aucPR) [66] to measure the classification accuracy. All the performance metrics were computed by following the five-fold cross-validation. For the classification using RF, the sensitivity, specificity, accuracy, MCC and F1-score were computed based on the majority voting scheme over 5 RF classifiers. On the other hand, the aucROC and aucPR were computed by taking average over the 5 RFs. Prediction for the independent test set was made by passing it through all the 9 binary classifiers, where each binary classifier was built with 5 RF classifiers. The prediction label for each test instance was decided based on the majority voting of the 5 RF classifiers.

## Results

### Feature generation and selection analysis

**Fig. 5:**
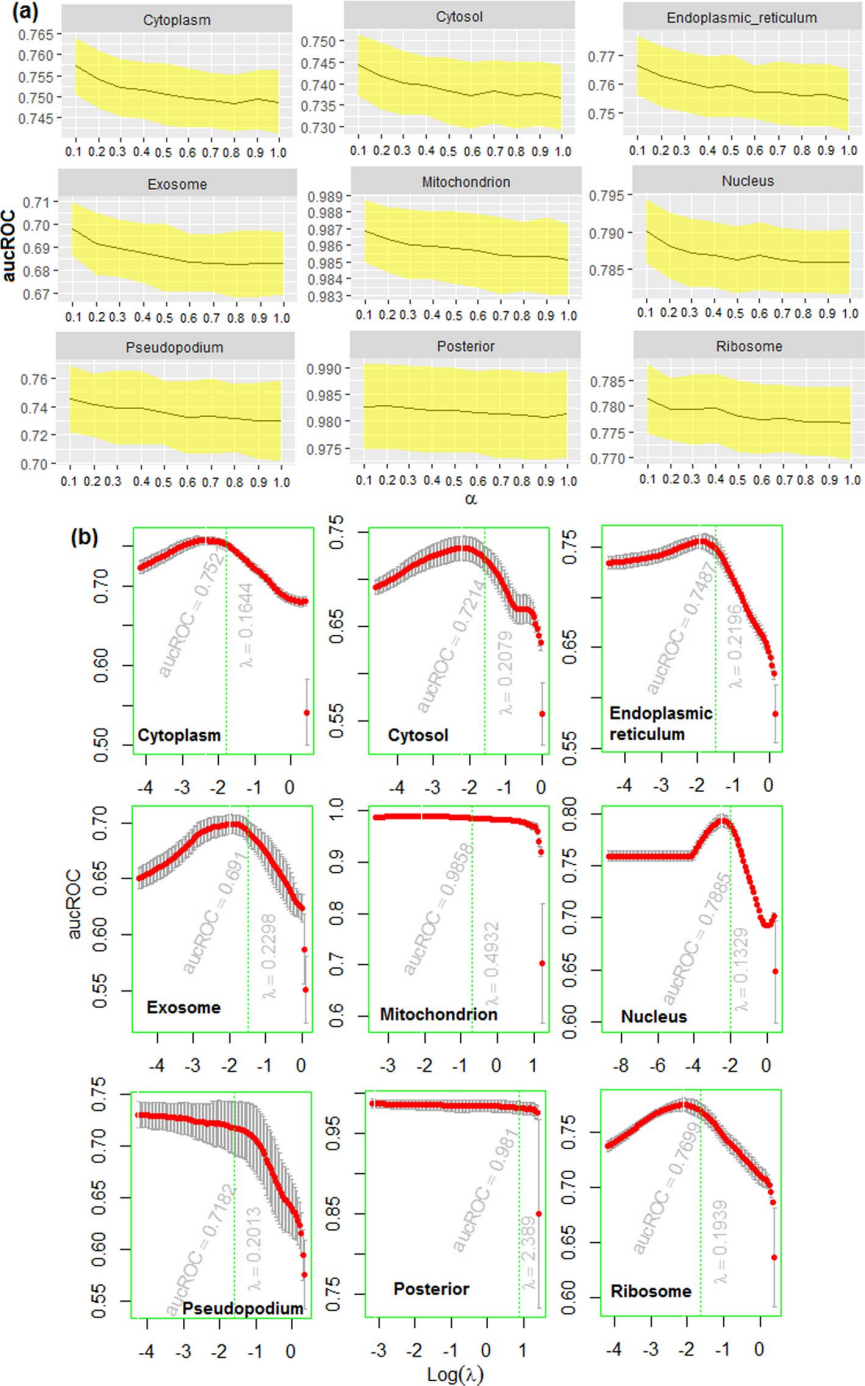
**a** Accuracies of the Elastic Net in terms of aucROC with respect to regularization parameter α. It is seen that the accuracies are decreased with increase in the value of α. Irrespective of the localization, the highest accuracy is obtained with α = 0.1, out of 10 different values of α (0.1, 0.2, 0.3, 0.4, 0.5, 0.6, 0.7, 0.8, 0.9, 1). **b** Trend in accuracy for Elastic Net with respect to λ. It is seen that the optimum value of λ is different for different localizations. Except posterior, the optimum value of λ is less than 1 for the remaining localizations

**Fig. 6:**
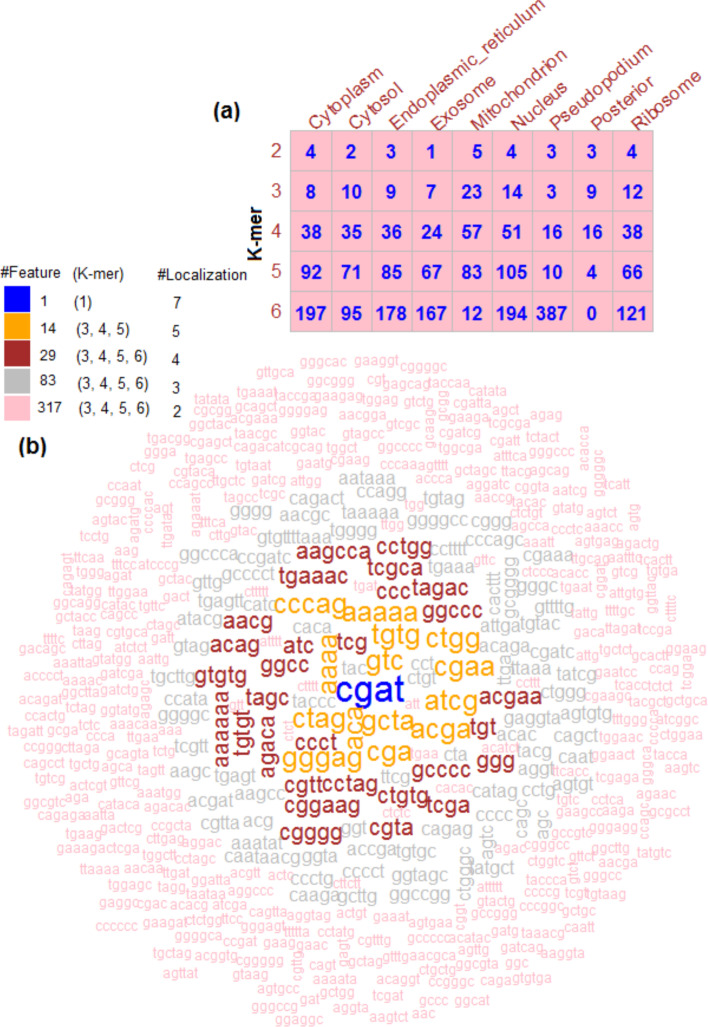
**a** Heat map showing the distribution of selected *k*-mer features in different localizations. **b** Cloud plotting of important *k*-mer features. It is observed that the feature CGAT of *k*-mer size 4 is the most important which is selected in 7 out of 9 localizations

Out of 10 different values of $\alpha$ in Elastic Net, higher aucROC were obtained for $\alpha =0.1$, irrespective of the localizations (Fig. 5a). Thus, the optimum value of $\alpha$ was determined as 0.1. With an increase in the value of $\alpha$, accuracy was declined and stabilized after $\alpha =0.8$ for all the localizations (Fig. 5a). Using $\alpha$=0.1, the optimum values of aucROC i.e., 0.752, 0.721, 0.748, 0.691, 0.985, 0.788 0.718, 0.981 and 0.769 were obtained with $\lambda$=0.1644, 0.2079, 0.2196, 0.2298, 0.4932, 0.1329, 0.2013, 2.389 and 0.1939 respectively for the corresponding localizations (Fig. 5b). With the determined optimum values of $\alpha$ and $\lambda$, the number of *k*-mer features with non-zero coefficients were observed to be 339, 213, 311, 266, 180, 368, 509, 32 and 241 for the respective localizations. Distribution of the selected features for different *k-*mer sizes is shown in Fig. 6a. For instance, the number of selected features for the cytoplasm with *k*-mer sizes 2, 3, 4, 5 and 6 were 4, 8, 38, 92 and 197 respectively. For *k*-mer size 1, even a single feature was not found with non-zero coefficient for all the 9 localizations. Out of 2459 selected features across localizations, 1812 were found to be non-redundant. The *k*-mer CGAT was observed as the most important feature among all the 1812 *k*-mers because it was selected in 7 out of the 9 localizations (Fig. 6b). Further, 14, 29, 83 and 317 *k*-mer features were selected in 5, 4, 3 and 2 localizations respectively and the remaining features were found to be important in one location only. It was also observed that 9 out of the top 15 *k*-mer features were of *k*-mer size 4 (Fig. 6b). The 1812 non-redundant *k*-mer features were employed for the localization prediction using RF.

### Cross-validated prediction analysis using random forest

**Fig. 7:**
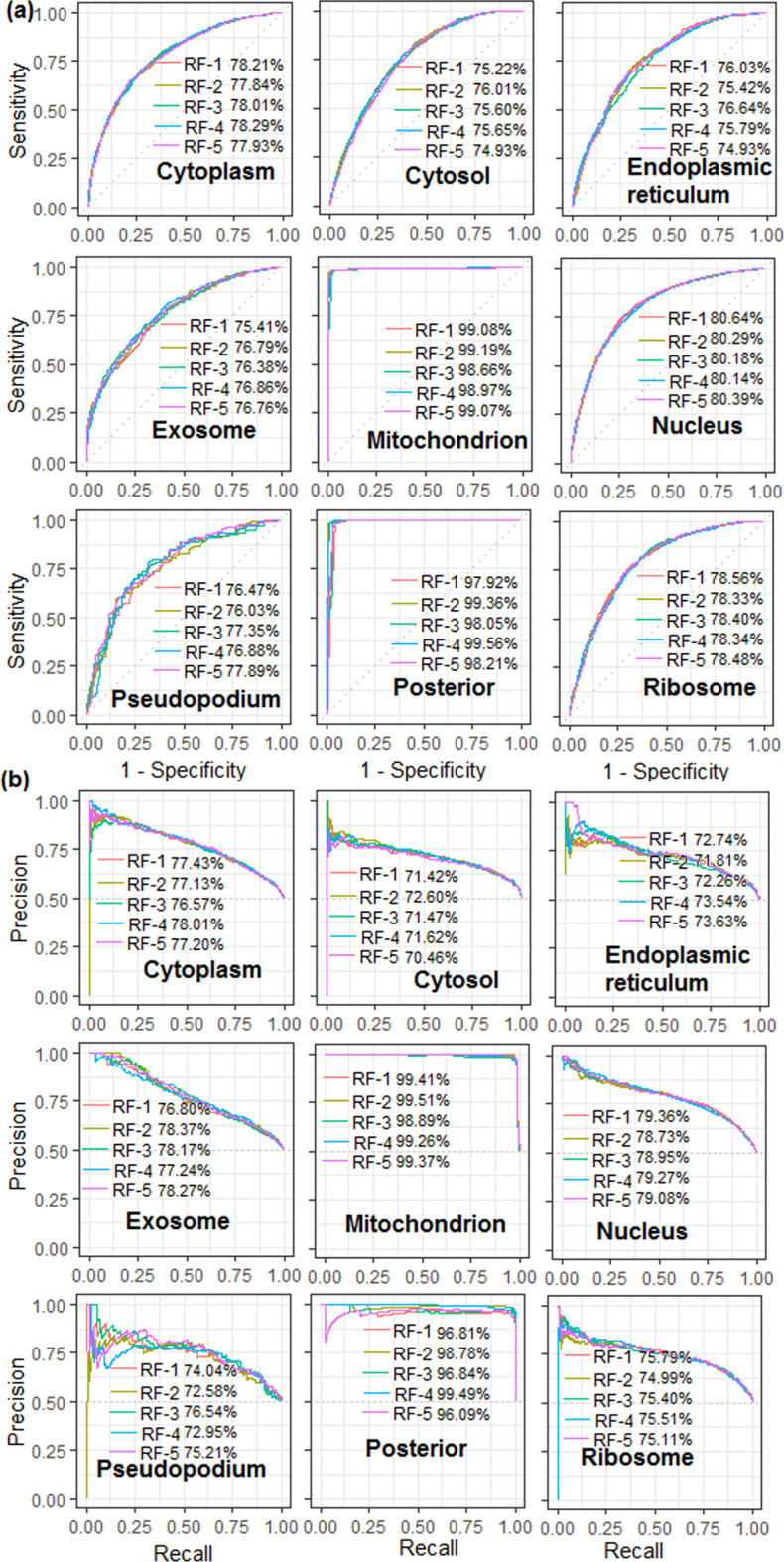
**a** Receiver operating characteristics (ROC) curves for predicting the localization of mRNAs using the Random Forest (RF) classification algorithm. In each localization, 5 RF classifiers in each localization. The dotted line represents the line of random guess which is 0.5 in the present scenario

Cross-validated performance metrics are presented in Table 1, whereas the ROC and PR curves for all the 5 RF classifiers are displayed in Fig. 7. Accuracies of 70.87, 68.32, 68.36, 68.79, 96.46, 73.44, 70.94, 97.42 and 71.77% were obtained for the respective localizations (Table 1). The average values of aucROC and aucPR over all the 5 RFs were observed to be ~ 75–98% and ~ 71–99% respectively (Table 1). The highest accuracy was found for the mitochondrion followed by the posterior, whereas the lowest accuracy was observed for the cytosol. Higher accuracies for the mitochondrion and posterior may be attributed to the fact that the mRNA sequences of these localizations were exclusively present in the respective localizations (Fig. 1a). More stable accuracies were observed for the larger size datasets (cytoplasm, cytosol, nucleus and ribosome) as compared to the smaller size datasets (endoplasmic reticulum, exosome, mitochondrion and pseudopodium). Although the sample size was small for the posterior, the accuracies were still found to be more stable as compared to the other localization with the small sample sizes. The probable reason may be that the sequences of the posterior are more conserved as compared to the other localizations which was evident from the fact that the sequences of the posterior were not present in other localizations (Fig. 1a).

**Table 1:** Performance metrics of mRNA localization prediction using random forest (RF)

Localization	Performance metrics						
	Sensitivity	Specificity	Accuracy	MCC	F1-score	aucROC	aucPR
Cytoplasm (1504)	73.24 ± 0.79	68.51 ± 1.36	70.87 ± 0.37	41.80 ± 0.71	71.53 ± 0.24	78.13 ± 0.20	77.43 ± 0.61
Cytosol (1798)	64.53 ± 0.61	72.11 ± 0.63	68.32 ± 0.16	36.75 ± 0.32	66.83 ± 0.56	75.63 ± 0.28	71.75 ± 0.49
EPR (850)	63.04 ± 1.72	73.68 ± 1.68	68.36 ± 0.99	36.95 ± 2.00	66.84 ± 1.27	75.54 ± 0.54	72.79 ± 0.77
Exosome (703)	63.20 ± 1.56	74.37 ± 1.13	68.79 ± 1.21	37.81 ± 2.41	66.74 ± 1.25	76.47 ± 0.62	77.57 ± 0.67
Mitochondrion (381)	98.53 ± 0.14	91.66 ± 7.95	96.46 ± 1.03	91.26 ± 5.76	95.59 ± 3.66	98.98 ± 0.20	99.27 ± 0.24
Nucleus (2754)	72.89 ± 0.92	73.99 ± 0.19	73.44 ± 0.54	46.88 ± 1.07	73.51 ± 0.60	80.28 ± 0.21	79.12 ± 0.27
Pseudopodium (180)	72.89 ± 1.20	69.00 ± 1.64	70.94 ± 0.97	41.93 ± 1.94	71.34 ± 1.02	76.73 ± 0.50	73.82 ± 1.62
Posterior (156)	98.19 ± 0.29	96.65 ± 0.84	97.42 ± 0.46	94.85 ± 0.90	97.19 ± 0.55	98.90 ± 0.83	98.29 ± 1.36
Ribosome (1532)	72.65 ± 1.01	70.89 ± 0.81	71.77 ± 0.24	43.55 ± 0.50	72.19 ± 0.51	78.40 ± 0.09	75.44 ± 0.29

For each localization, a balanced dataset with equal number of positive and negative instances was used for prediction using RF. Performance metrics are computed following majority voting strategy, where 5 RF classifiers are constructed for each localization. Besides, accuracies in each classifier are measured following five-fold cross-validation

*EPR* Endoplasmic reticulum

### Comparative analysis with other bootstrapping methods

**Fig. 8:**
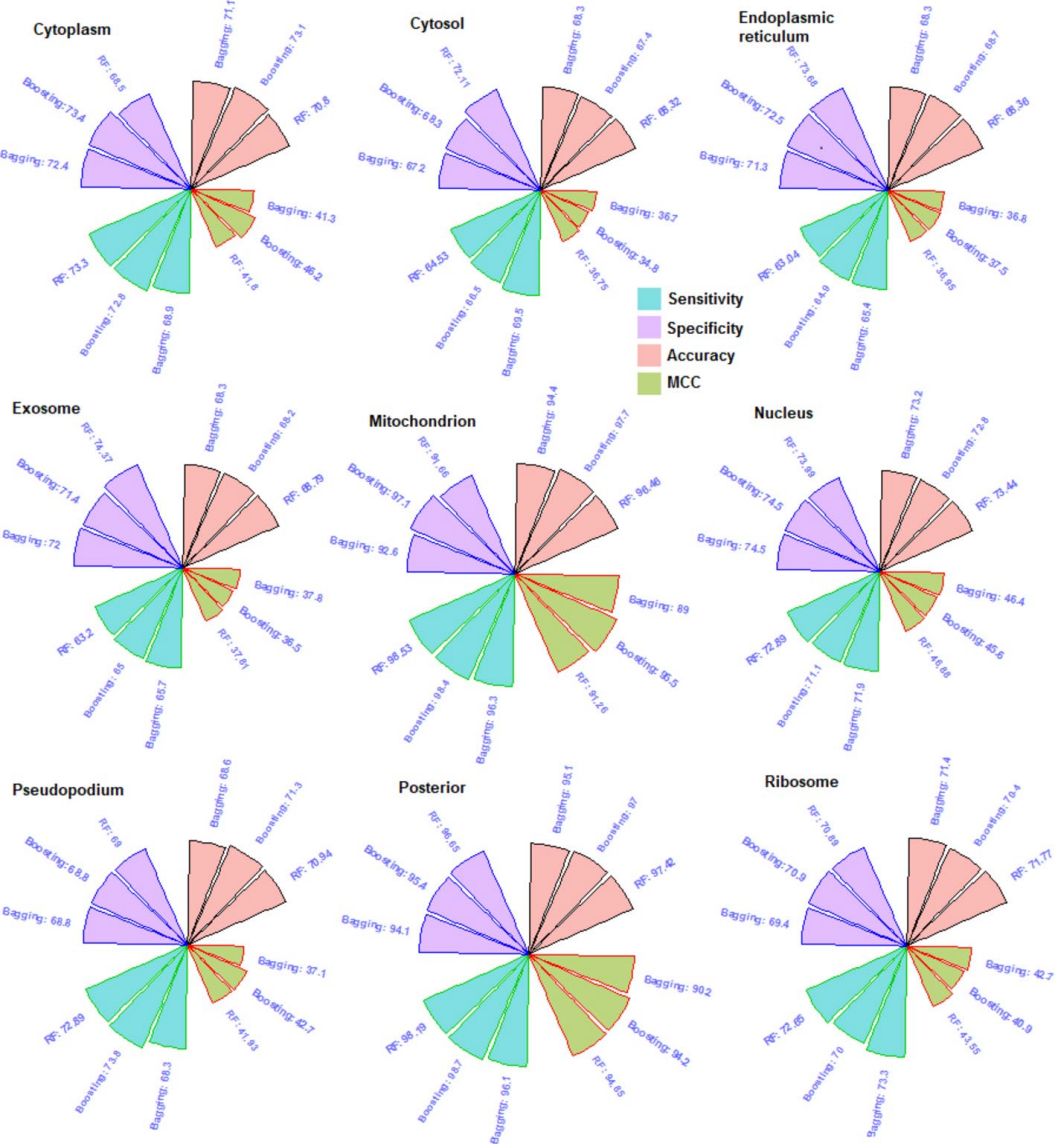
Circular bar plots of the performance metrics of RF, Bagging and Boosting algorithms. The accuracy and MCC of the RF are higher in five localizations (cytosol, exosome, nucleus, posterior, ribosome), whereas the Boosting algorithm performed better in the remaining four localizations (cytoplasm, endoplasmic reticulum, mitochondrion, pseudopodium). The Bagging classifier achieved the lowest accuracy and MCC than that of RF and Boosting classifiers

We have compared the performance of RF with other bootstrapping methods i.e., Bagging [67] and Boosting [68]. Performances were computed in the same way as done in the case of RF, by using the same dataset. The Bagging and Boosting algorithms were implemented by using the *ipred* [69] and *adabag* [70] R-packages respectively. Default parameters setting were employed for prediction. The results are shown in Fig. 8. Accuracies of the RF for the cytosol (68.32), exosome (68.79), nucleus (73.44), posterior (97.42) and ribosome (71.77) were higher than that of Bagging (68.3, 68.3, 73.2, 95.1, 71.4) and Boosting (67.4, 68.2, 72.8, 97.0, 70.4). On the other hand, the accuracies of the Boosting algorithm were higher for the cytoplasm (73.1), endoplasmic reticulum (68.7), mitochondrion (97.7) and pseudopodium (71.3) than that of Bagging (71.1, 68.3, 94.4, 68.6) and RF (70.87, 68.36, 96.46, 70.94) methods. Similarly, the values of MCC of the RF were higher for the localizations cytosol (36.75), exosome (37.81), nucleus (46.88), posterior (94.85) and ribosome (43.55) than that of Bagging (36.7, 37.8, 46.4, 90.2, 42.7) and Boosting (34.8, 36.5, 45.6, 94.2, 40.9). On the other hand, Boosting algorithm achieved higher MCC for the cytoplasm (46.2), endoplasmic reticulum (37.5), mitochondrion (95.5) and pseudopodium (42.7) than that of Bagging (41.3, 36.8, 89.0, 37.1) and RF (41.8, 36.95, 91.26, 41.93) methods.

### Performance analysis of random forest with different *k*-mer features

**Fig. 9:**
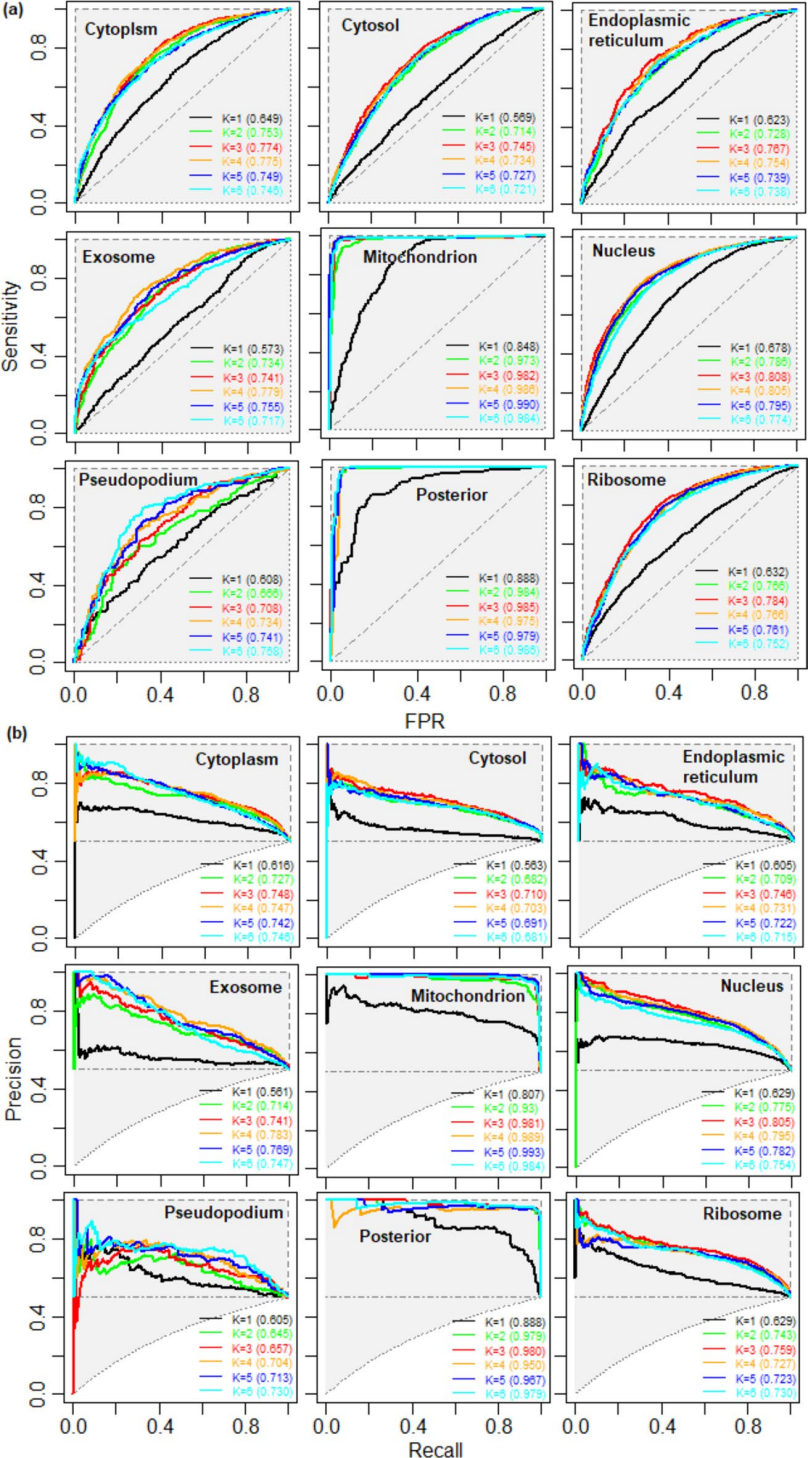
**a** Receiver operating characteristics (ROC) curves and **b** precision-recall curves for the RF classifier with different *k*-mer features

To study the effect of different *k*-mer sizes, the performance of RF was analyzed with six different *k*-mer features i.e., *k* = 1, *k* = 2, *k* = 3, *k* = 4, *k* = 5 and *k* = 6. Thus, the numbers of features utilized were 4, 16, 64, 256, 1024 and 4096 respectively. The accuracies were measured in terms of aucROC and aucPR following five-fold cross validation (Fig. 9). Except exosome, mitochondrion and pseudopodium, the accuracies are increased up to *k* = 3 for rest of the 6 localizations, whereas the accuracies for the exosome, mitochondrion and pseudopodium are found to be increased upto *k* = 4, *k* = 5 and *k* = 6 respectively (Fig. 9). Higher accuracy with *k* = 3 may be attributed to the formation of codon structure that resulted in better discrimination of mRNA localizations. On the other hand, less accuracy with *k*-mer sizes 4, 5 and 6 may be due to the generation of redundant features (mostly contained 0 s). This implies that the model performance may not increase with further increment in the *k*-mer size.

### Evaluation with independent test set-I

Classification accuracies for the independent test set-I were obtained as 65.33, 71.37, 75.86, 72.99, 94.26, 70.91, 65.53, 93.60 and 73.45% respectively (Table 2). The aucROC values (~ 73–98%) were observed at par with the cross-validation accuracy, whereas the aucPR values (~ 7–75%) were much lower than that of cross-validation accuracy because of the highly imbalanced test dataset (Table 2). This is because ROC is independent of the class distribution and PR is dependent upon the number of observations present in different classes. The MCC also takes into account the class distribution and hence its values were also observed to be less because of imbalanced dataset (Table 2).

**Table 2:** Performance metrics for the Independent test set-I

Localization	Performance metrics						
	Sensitivity	Specificity	Accuracy	MCC	F1-measure	aucROC	aucPR
Cytoplasm (300)	70.00	64.49	65.33	25.22	38.09	74.72	37.88
Cytosol (360)	64.17	72.98	71.37	30.35	45.04	76.30	40.51
EPR (170)	94.59	74.71	75.86	35.62	40.93	73.57	17.80
Exosome (140)	66.43	73.50	72.99	22.53	25.92	76.49	34.84
Mitochondrion (76)	82.89	94.72	94.26	54.26	52.73	95.81	75.60
Nucleus (550)	75.09	69.30	70.91	40.21	59.06	78.75	57.96
Pseudopodium (36)	55.56	65.72	65.53	5.99	5.57	69.56	6.55
Posterior (31)	100.00	93.50	93.60	42.97	32.98	98.45	44.12
Ribosome (306)	66.34	74.76	73.45	32.01	43.72	77.91	37.67

Prediction for the test set was made by passing it through all the 9 binary classifiers corresponding to 9 localizations. The accuracies are found to be consistent with that of training set, except aucPR. The low aucPR may be attributed to the highly unbalanced nature of the test dataset, where for a given location sequences of the remaining locations together constituted the negative set

*EPR* Endoplasmic reticulum

### Evaluation with independent test set-II

Performance metrics for the independent test set-II are given in Table 3. Accuracies for the respective localizations were observed to be 69.34, 75.91, 74.91, 78.10, 95.28, 80.91, 93.16 and 78.69% respectively. Prediction accuracies were also found to be higher than that of independent test set-I, because independent test set-II shares a higher degree of similarity with the training set. The aucROC values were also much higher and it may be because ROC does not account for the class distribution.

**Table 3:** Prediction accuracies with Independent test set-II

Localization	Performance metrics						
	Sensitivity	Specificity	Accuracy	MCC	F1-score	aucROC	aucPR
Cytoplasm (490)	91.84	66.57	69.34	37.26	39.66	91.77	73.72
Cytosol (1037)	89.78	71.73	75.91	52.49	63.39	92.21	83.36
EPR (485)	91.55	72.88	74.91	42.25	44.21	92.00	69.50
Exosome (185)	88.65	77.65	78.10	30.44	25.12	92.29	64.38
Mitochondrion (14)	92.86	95.29	95.28	22.62	10.99	98.97	49.99
Nucleus (1266)	90.76	76.47	80.51	61.25	72.54	93.00	86.41
Pseudopodium (79)	93.67	69.02	69.45	17.68	9.79	92.93	59.72
Posterior (121)	100.00	92.97	93.16	51.33	44.20	98.11	53.66
Ribosome (789)	91.48	73.73	76.89	51.45	58.28	93.05	80.26

The sensitivities are found to be much higher than that of Independent test set-I. Because, this dataset shares > 80% sequence similarity with the training set. However, overall accuracies are found at par with the training dataset. It can also be seen that the aucROC values are much higher, may be due to higher degree of sequence similarity with the training set

*EPR* Endoplasmic reticulum

### Comparative analysis with the existing methods

There are three tools such as RNATracker [34], iLoc-mRNA [35] and mRNALoc [36] available for predicting mRNA localizations. Since the RNATracker has been trained with the CeFra-Seq/APEX-RIP dataset involving gene expression and coordinate files, it was not included for comparison. Between iLoc-mRNA and mRNALoc, the latter one has been shown to outperform the former one. Thus, we compared with the mRNALoc which is also the latest one in the series. The comparison was made using two datasets, i.e., Independent test set-I (Test set-I) and independent dataset of mRNALoc (Test set-II). It was also ensured that sequences of the mRNALoc test set were not present in our training set and sequences of the Independent test set-I were not present in the training set of mRNALoc. Furthermore, the comparison was made with the 4 localizations (cytoplasm, endoplasmic reticulum, mitochondrion and nucleus) that were common between the mRNALoc and our study. The performance metrics were computed by considering the sequences of a given localization as the positive set and the remaining three localizations as the negative set. For instance, in test set-I, the number of positive instances for the nucleus was 83 and the number of negative instances was 142 (86 + 31 + 25). For the test set-I, the proposed approach achieved higher accuracies (67.9, 60.7) and MCC (36.1, 23.8) for the localizations cytoplasm and nucleus respectively (Table 4). On the other hand, mRNALoc achieved higher accuracies (70.2, 70.9) and MCC (42.1, 47.5) for the localizations endoplasmic reticulum and mitochondrion respectively (Table 4). For the test set-II, the developed approach achieved higher accuracies for all the four localizations i.e., cytoplasm (64.3), endoplasmic reticulum (83.0), mitochondrion (91.3) and nucleus (77.6). Except for mitochondrion, the MCC values of the proposed approach were also higher for the rest of the three localizations i.e., cytoplasm (28.6), mitochondrion (62.4) and nucleus (21.4). Taking both the test sets into account, the proposed approach may be said to perform better than that of mRNALoc.

**Table 4:** Comparative analysis of the developed approach with the mRNALoc

	mRNALoc					Proposed				
	Sn	Sp	Ac	F1-score	MCC	Sn	Sp	Ac	F1-score	MCC
*Test set-I*										
Cytoplasm (86)	39.5	75.6	57.5	44.1	16.2	76.7	59.0	**67.9**	**63.1**	**36.1**
Endoplasmic reticulum (31)	51.7	88.8	70.2	46.6	42.1	44.8	91.8	68.3	45.7	40.2
Mitochondrion (25)	48.0	93.8	70.9	48.6	47.5	56.0	73.5	64.8	30.4	22.3
Nucleus (83)	50.0	62.2	56.1	46.1	12.3	39.0	82.3	**60.7**	**46.6**	**23.8**
*Test set-II*										
Cytoplasm (464)	52.6	64.5	58.6	52.6	17.2	63.4	65.3	**64.3**	**60.5**	**28.6**
Endoplasmic reticulum (103)	43.7	78.4	61.0	25.0	17.3	72.8	93.1	**83.0**	**61.2**	**62.4**
Mitochondrion (8)	75.0	97.8	86.4	65.2	47.7	100	82.7	**91.3**	31.6	21.4
Nucleus (508)	55.5	78.7	67.1	61.8	34.4	55.1	100	**77.6**	**71.1**	**58.6**

For the Test set-I, the developed method achieved higher accuracy than the mRNALoc for cytoplasm and nucleus localizations. For the Test set-II, the developed method performed better than mRNALoc in all the four localizations

Bold font denotes the higher performance of the proposed approach as compared to the mRNALoc

*Sn* sensitivity, *Sp* specificity, *Ac* accuracy, *MCC* Matthew’s correlation coefficient

### Prediction server mLoc-mRNA

**Fig. 10:**
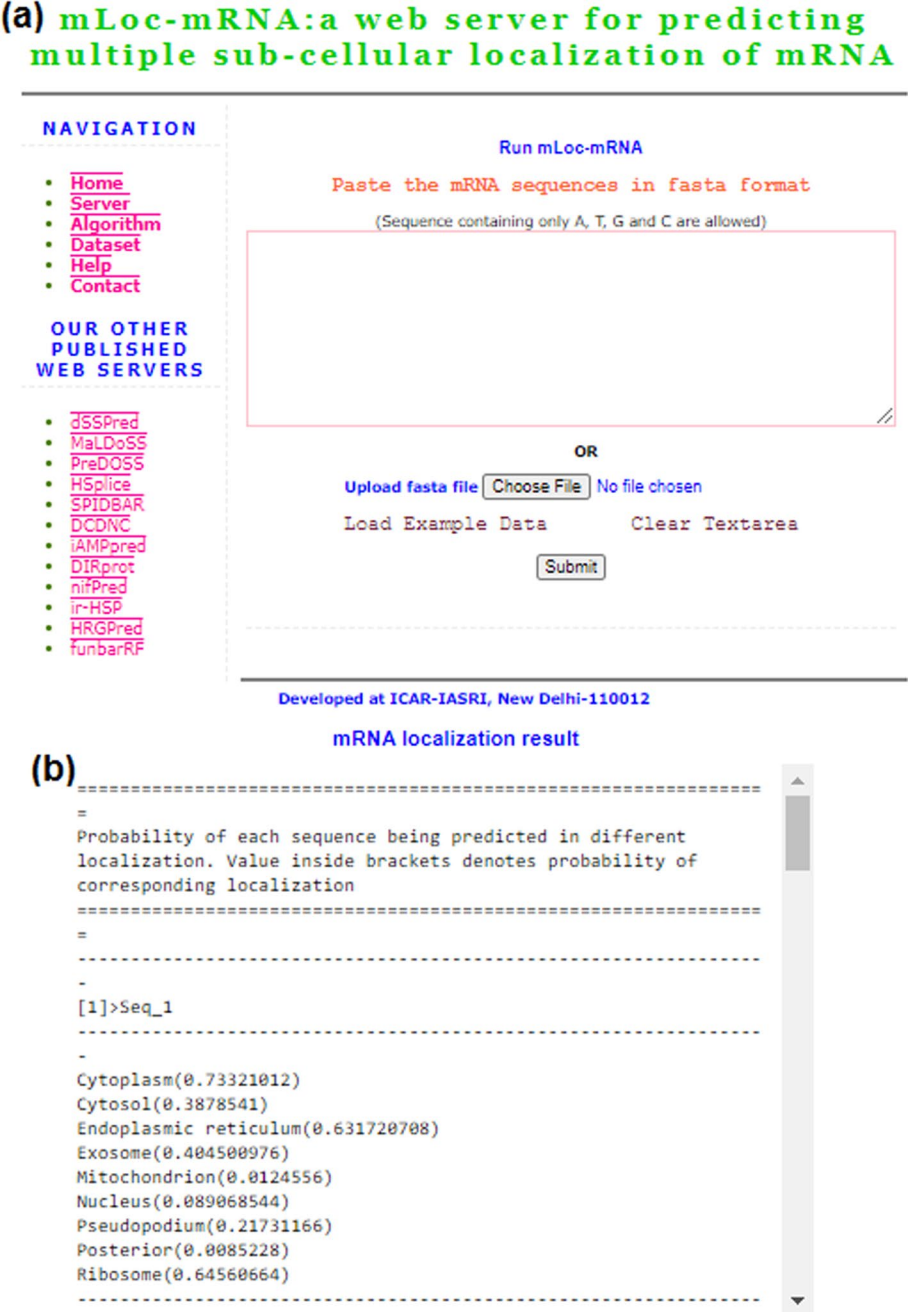
**a** Diagram showing the server page of mLoc-mRNA. The user can either submit the sequence file in FASTA format or upload the sequences in the text area for prediction. **b** Prediction result for an example sequence, where the probability of prediction in each localization is provided

An online prediction server “mLoc-mRNA” is freely accessible at http://cabgrid.res.in:8080/mlocmrna/ for predicting the multiple subcellular localization of mRNAs. The front-end of the server has been designed by using HTML (hypertext markup language), whereas the developed R-programs run at the back end with the help of PHP (hypertext preprocessor). There are provisions for both uploading the FASTA file of mRNA sequences as well as pasting of mRNA sequences in the text area by the user (Fig. 10a). The results were shown in terms of probabilities with which each mRNA sequence was predicted in 9 different localizations (Fig. 10b). The value 0.5 was treated as a threshold above which the test instance was predicted in the positive class and the negative class otherwise.

## Discussion

Localization of mRNAs is an evolutionarily conserved phenomenon, found in plants, animals as well as in unicellular organisms [8]. Coupled with the translational regulation, subcellular localization of mRNAs provides means to control the time and location of the protein synthesis and their functions [2, 3, 8, 28]. Genome-wide association analysis [71–73] suggests that the establishment of functionally distinct compartments happens through sub-cellular targeting of mRNAs by polarized cells. Keep in view the numerous advantages of mRNA localization, this study presents a new computational model for predicting the multiple subcellular localization of mRNAs.

The mRNA localization datasets were collected from the RNALocate database. Besides the considered 9 localizations, mRNA sequences were also available for other localizations such as anterior, apical, axon and others. However, after removing the overlapped and redundant sequences, very few sequences were left for these localizations and thus not considered in this study. Though the cytosol is the aqueous part of the cytoplasm, it has its own function i.e., cytosol concentrates the dissolved molecules into the correct position for efficient metabolism, and a large number of proteins are localized in the cytosol [74]. Therefore, both cytoplasm and cytosol were considered despite the fact that the cytosol is the aqueous part of the cytoplasm. Furthermore, there were overlapped sequences between these two localizations, and we utilized the dataset after removing not only the overlapped sequences but also the sequences that shared more than 80% sequence identity with any other sequences in each localization.

We utilized *k*-mer features for translating the nucleotide sequences into numeric vectors, where the important *k*-mer features were selected by using the Elastic Net model. The LASSO eliminates the redundant/irrelevant features and hence reduces the over-fitting of the model. On the other hand, Ridge assigns less weight to the features that are less relevant for predicting the response. Elastic Net combines the features of both LASSO and the Ridge to improve the prediction performance [39]. Thus, the Elastic Net may be more effective as compared to the LASSO and Ridge. Hence, we employed the Elastic Net for the feature selection.

The RF algorithm was employed for the prediction purpose because it is an ensemble learning method that tends to produce higher accuracy as compared to the single base classifier [40]. The performance of the developed computational model was evaluated by following a five-fold cross-validation procedure as well as by using the independent test sets. Higher accuracies were obtained for the mitochondrion and posterior as compared to the other localizations because the sequences of these two localizations share a lesser degree of similarity with other localizations which is evident from the fact that the sequences of these localizations were present in the remaining localizations (Fig. 1a). The RF model is not only useful for the classification/prediction but also for the selection of important features through RF variable importance measure. There are two types of RF variable importance measure i.e., mean decrease in impurity (Gini importance) and mean decrease in accuracy (permutation importance). For the permutation importance of a variable, the prediction accuracy on the OOB sample is first computed. Then, the values of the variable in the OOB samples are permuted keeping all other variables the same and the accuracy with the permuted data is again computed. The difference in the prediction accuracy caused by the permutation is measured and averaged over all the tree classifiers of RF. The idea of permutation importance is that if a variable is important to achieve high prediction accuracy, shuffling that variable should lead to an increase in the error and vice-versa. Mean decrease in impurity is another variable importance measure of RF. The Gini impurity is used as a measure to choose the variable for splitting of parental node into daughter nodes. For a particular variable, the decrease in Gini impurity is accumulated for each tree every time the variable is chosen to split a node. The sum is averaged over all the trees in the forest to get the importance of each such variable. Though the Gini importance is a biased measure, minimal computation is required for the Gini impurity. On the other hand, though permutation-based importance employs an exhaustive permutation, it is a reasonably efficient technique than that of Gini importance.

Besides RF, we have also used the ensemble learning methods Bagging and Boosting. In terms of accuracy and MCC, Bagging achieved less accuracy than that of RF and Boosting methods. Out of 9 localizations, RF achieved higher accuracy and MCC in 5 localizations whereas Boosting achieved higher accuracy and MCC in the other four localizations. In other words, RF achieved higher accuracy in majority of the localization. However, the difference in accuracies between RF and Boosting algorithm were not much higher. Further, Boosting algorithm is more sensitive to noisy data and over fitting as compared to the RF because the classification trees are grown independently in RF whereas the classification trees are correlated in Boosting. Also, less numbers of parameters are needed to be tuned in RF as compared to the Boosting algorithm. Keeping all the above points in mind, we preferred RF for prediction in all the 9 localizations. In Bagging, randomization is only introduced while drawing the bootstrap sample, whereas in RF randomization is introduced at two stages i.e., during drawing the bootstrap sample and splitting of the nodes of the classification tree [40]. This may be the reason RF achieved higher accuracy than that of Bagging. As far as Boosting method is concerned, it works sequentially and iteratively adjusts the weights of observations until a better learner is achieved. On the other hand, in Bagging each classification tree is grown independently without adjusting the weights of observations [75]. This may be the reason for the better accuracy of the Boosting over Bagging method. Since RF was preferred for prediction in 9 localizations as compared to other bootstrapping methods, the RF was only used for prediction of the independent dataset. The cross validation accuracy and the accuracy with the independent dataset were found to be similar for the developed approach.

Performance of the mLoc-mRNA was also compared with the state-of-art single localization prediction tool mRNALoc using two different independent datasets. The mLoc-mRNA achieved higher accuracy in two localizations with the test set-I and all the four localizations with the test set-II. Thus, the proposed approach may achieve higher accuracy than that of mRNALoc with regard to predicting the single localization of mRNAs. The performance was compared with that of mRNALoc only because it has outperformed the other existing methods such as RNATracker [34] and iLoc-mRNA [35]. We have used the localization dataset of the RNALocate database that contains data from 65 organisms with most of the data from the model organisms like, *Homo sapiens*, *Mus musculus* and *Saccharomyces cerevisiae*. On the other hand, the existing tools such as RNATracker and iLoc-mRNA have been developed by using only localization data of human mRNA. Thus, the developed tool mLoc-mRNA is a more general one with regard to predicting the eukaryotic mRNA subcellular localization. Besides, the datasets i.e., CeFra-Seq [36], APEX-RIP [37] used in the RNATracker are sometimes noisy and inaccurate [38]. On the other hand, the dataset used in our study was collected from the RNALocate which contains a manually curated mRNA localization dataset with experimental evidence. Further, existing localization prediction tools such as RNATracker, iLoc-mRNA and mRNALoc have been developed for predicting the single localization of mRNAs. On the other hand, the mLoc-mRNA is meant for predicting both single as well as multiple subcellular localizations.

We have also developed an online prediction tool mLoc-mRNA (http://cabgrid.res.in:8080/mlocmrna/) for multiple localization prediction of mRNAs. Using the tool, the prediction label for the test instance is decided with the majority voting by 5 RF classifiers. The developed tool is expected to supplement the existing single localization prediction tools as well as the in situ hybridization technique for mRNA localization study.

## Conclusions

In this article, we have developed a new computational method for predicting multiple subcellular localization of mRNAs. By using the developed method, user can easily predict the probability of any mRNA sequence being predicted in 9 different localizations. The developed web server is expected to be of great help for researchers from the non-computational background. The proposed methodology will certainly supplement the existing studies towards the localization prediction of mRNAs.

## Supplementary Information

**Additional file1: Table S1**. Summary of the training data set, independent test set-I and II and comparison test set-I and II.

## Data Availability

All the datasets used in this study are available at http://cabgrid.res.in:8080/mlocmrna/dataset.html.

## Abbreviations

RNA: Ribonucleic acid
mRNA: Messenger RNA
UTR: Untranslated region
TIRF: Total internal reflection microscopy
SIM: Structured illumination microscopy
RF: Random forest
OOB: Out-of-bag
LASSO: Least absolute shrinkage and selection operator
ROC: Receiver operating characteristics
PR: Precision-recall
aucROC: Area under ROC curves
aucPR: Area under PR curves
HTML: Hypertext markup language
PHP: Hypertext preprocessor

## References

[1] Jeffery WR, Tomlinson CR, Brodeur RD (1983). Localization of actin messenger RNA during early ascidian development. Dev Biol.

[2] Holt CE, Bullock SL (2009). Subcellular mRNA localization in animal cells and why it matters. Science.

[3] Medioni C, Mowry K, Besse F (2012). Principles and roles of mRNA localization in animal development. Development.

[4] Weatheritt RJ, Gibson TJ, Babu MM (2014). Asymmetric mRNA localization contributes to fidelity and sensitivity of spatially localized systems. Nat Struct Mol Biol.

[5] Lazzaretti D, Bono F (2017). mRNA localization in metazoans: a structural perspective. RNA Biol.

[6] Teimouri H, Korkmazhan E, Stavans J, Levine E (2017). Sub-cellular mRNA localization modulates the regulation of gene expression by small RNAs in bacteria. Phys Biol.

[7] Martin KC, Ephrussi A (2009). mRNA localization: gene expression in the spatial dimension. Cell.

[8] Tian L, Chou HL, Fukuda M (2020). mRNA localization in plant cells. Plant Physiol.

[9] Di Liegro CM, Schiera G, Di Liegro I (2014). Regulation of mRNA transport, localization and translation in the nervous system of mammals. Int J Mol Med.

[10] Wang ET, Taliaferro JM, Lee JA (2016). Dysregulation of mRNA localization and translation in genetic disease. J Neurosci.

[11] Wang DO, Martin KC, Zukin RS (2010). Spatially restricting gene expression by local translation at synapses. Trends Neurosci.

[12] Mauger DM, Siegfried NA, Weeks KM (2013). The genetic code as expressed through relationships between mRNA structure and protein function. FEBS Lett.

[13] Jung H, Gkogkas CG, Sonenberg N (2014). Remote control of gene function by local translation. Cell.

[14] Cody NA, Iampietro C, Lécuyer E (2013). The many functions of mRNA localization during normal development and disease: from pillar to post. Wires Dev Biol.

[15] Fallini C, Donlin-Asp PG, Rouanet JP (2016). Deficiency of the survival of motor neuron protein impairs mRNA localization and local translation in the growth cone of motor neurons. J Neurosci.

[16] Chin A, Lécuyer E (2017). RNA localization: making its way to the center stage. Biochimica et Biophysica Acta (BBA).

[17] Hervé C, Mickleburgh I, Hesketh J (2004). Zipcodes and postage stamps: mRNA localization signals and their trans-acting binding proteins. Brief Funct Genom.

[18] Besse F, Ephrussi A (2008). Translational control of localized mRNAs: restricting protein synthesis in space and time. Nat Rev Mol Cell Biol.

[19] Meignin C, Davis I (2010). Transmitting the message: intracellular mRNA localization. Curr Opin Cell Biol.

[20] Tian L, Okita TW (2014). mRNA-based protein targeting to the endoplasmic reticulum and chloroplasts in plant cells. Curr Opin Plant Biol.

[21] Kloc M, Zearfoss NR, Etkin LD (2002). Mechanisms of subcellular mRNA localization. Cell.

[22] Doyle M, Kiebler MA (2012). A zipcode unzipped. Genes Dev.

[23] Mingle LA, Okuhama NN, Shi J (2005). Localization of all seven messenger RNAs for the actin-polymerization nucleator Arp2/3 complex in the protrusions of fibroblasts. J Cell Sci.

[24] Andreassi C, Riccio A (2009). To localize or not to localize: mRNA fate is in 3′UTR ends. Trends Cell Biol.

[25] Jung H, Yoon BC, Holt CE (2012). Axonal mRNA localization and local protein synthesis in nervous system assembly, maintenance and repair. Nat Rev Neurosci.

[26] Buxbaum AR, Wu B, Singer RH (2014). Single β-actin mRNA detection in neurons reveals a mechanism for regulating its translatability. Science.

[27] Little SC, Tkačik G, Kneeland TB (2011). The formation of the Bicoid morphogen gradient requires protein movement from anteriorly localized mRNA. PLoS Biol.

[28] Parton RM, Davidson A, Davis I, Weil TT (2014). Subcellular mRNA localization at a glance. J Cell Sci.

[29] Wu B, Chao JA, Singer RH (2012). Fluorescence fluctuation spectroscopy enables quantitative imaging of single mRNAs in living cells. Biophys J.

[30] Sinsimer KS, Lee JJ, Thiberge SY (2013). Germ plasm anchoring is a dynamic state that requires persistent trafficking. Cell Rep.

[31] Weil TT, Parton RM, Davis I (2010). Making the message clear: visualizing mRNA localization. Trends Cell Biol.

[32] Altschul SF, Gish W, Miller W, Myers EW, Lipman DJ (1990). Basic local alignment search tool. J Mol Biol.

[33] Johnson LS, Eddy SR, Portugaly E (2010). Hidden Markov model speed heuristic and iterative HMM search procedure. BMC Bioinform.

[34] Yan Z, Lecuyer E, Blanchette M (2019). Prediction of mRNA subcellular localization using deep recurrent neural networks. Bioinformatics.

[35] Zhang ZY, Yang YH, Ding H (2020). Design powerful predictor for mRNA subcellular location prediction in Homo sapiens. Brief Bioinform.

[36] Garg A, Singhal N, Kumar R, Kumar M (2020). mRNALoc: a novel machine-learning based in-silico tool to predict mRNA subcellular localization. Nucleic Acids Res.

[37] Bouvrette LPB, Cody NA, Bergalet J (2018). CeFra-seq reveals broad asymmetric mRNA and noncoding RNA distribution profiles in Drosophila and human cells. RNA.

[38] Kaewsapsak P, Shechner DM, Mallard W (2017). Live-cell mapping of organelle-associated RNAs via proximity biotinylation combined with protein-RNA crosslinking. Elife.

[39] Zou H, Hastie T (2005). Regularization and variable selection via the elastic net. J R Stat Soc Ser B Stat Methodol.

[40] Breiman L (2001). Random forests. Mach Learn.

[41] Zhang T, Tan P, Wang L (2017). RNALocate: a resource for RNA subcellular localizations. Nucleic Acids Res.

[42] Huang Y, Niu B, Gao Y (2010). CD-HIT Suite: a web server for clustering and comparing biological sequences. Bioinformatics.

[43] Su ZD, Huang Y, Zhang ZY (2018). iLoc-lncRNA: predict the subcellular location of lncRNAs by incorporating octamer composition into general PseKNC. Bioinformatics.

[44] Melsted P, Pritchard JK (2011). Efficient counting of k-mers in DNA sequences using a bloom filter. BMC Bioinform.

[45] Han GB, Cho DH (2019). Genome classification improvements based on k-mer intervals in sequences. Genomics.

[46] Manekar SC, Sathe SR (2018). A benchmark study of *k*-mer counting methods for high-throughput sequencing. GigaScience.

[47] Zhu PP, Li WC, Zhong ZJ (2015). Predicting the subcellular localization of mycobacterial proteins by incorporating the optimal tripeptides into the general form of pseudo amino acid composition. Mol BioSyst.

[48] Zhao YW, Su ZD, Yang W (2017). IonchanPred 20: a tool to prediction channels and their types. Int J Mol Sci.

[49] Feng P, Yang H, Ding H (2019). iDNA6mA-PseKNC: identifying DNA N6-methyladenosine sites by incorporating nucleotide physicochemical properties into PseKNC. Genomics.

[50] Yang H, Tang H, Chen XX et al. Identification of secretory proteins in mycobacterium tuberculosis using pseudo amino acid composition. Biomed Res Int. 2016:1 10.1155/2016/5413903 PMC4997101 27597968

[51] Liu B, Fang L, Wang S (2015). Identification of microRNA precursor with the degenerate K-tuple or Kmer strategy. J Ther Biol.

[52] Lai HY, Chen XX, Chen W (2017). Sequence-based predictive modeling to identify cancerlectins. Oncotarget.

[53] Tibshirani R (1996). Regression shrinkage and selection via the lasso. J R Stat Soc Ser B Stat Methodol.

[54] Hoerl AE, Kannard RW, Baldwin KF (1975). Ridge regression: some simulations. Commun Stat Theory Methods.

[55] Friedman J, Hastie T, Tibshirani R (2009). glmnet: lasso and elastic-net regularized generalized linear models. R Pack Ver.

[56] Díaz-Uriarte R, Azuaje F, Dopazo J (2005). Supervised methods with genomic data: a review and cautionary view. Data analysis and visualization in genomics and proteomics.

[57] Hua J, Xiong Z, Lowey J (2005). Optimal number of features as a function of sample size for various classification rules. Bioinformatics.

[58] Meher PK, Sahu TK, Rao AR (2016). Prediction of donor splice sites using random forest with a new sequence encoding approach. BioData Min.

[59] Chen X, Ishwaran H (2012). Random forests for genomic data analysis. Genomics.

[60] Liaw A, Wiener M (2002). Classification and regression by randomForest. Rnews.

[61] Cheng X, Xiao X, Chou KC (2018). pLoc-mEuk: predict subcellular localization of multi-label eukaryotic proteins by extracting the key GO information into general PseAAC. Genomics.

[62] Liu B, Yang F, Huang DS (2018). iPromoter-2L: a two-layer predictor for identifying promoters and their types by multi-window-based PseKNC. Bioinformatics.

[63] Meher PK, Sahu TK, Saini V (2017). Predicting antimicrobial peptides with improved accuracy by incorporating the compositional, physico-chemical and structural features into Chou’s general PseAAC. Sci Rep.

[64] Meher PK, Sahu TK, Gahoi S, Rao AR (2018). ir-HSP: improved recognition of heat shock proteins, their families and sub-types based on g-spaced di-peptide features and support vector machine. Front Genet.

[65] Fawcett T (2006). An introduction to ROC analysis. Pattern Recognit Lett.

[66] Davis J, Goadrich M. The relationship between Precision-Recall and ROC curves. In: Proceedings of the 23rd international conference on machine learning. 2006, pp 233–240

[67] Breiman L. Bagging predictors. Technical Report 421, Department of Statistics, UC Berkeley (1994)

[68] Drucker H, Cortes C, Jackel LD, LeCun Y, Vapnik V (1994). Boosting and other ensemble methods. Neural Comput.

[69] Peters A, Hothorn T, Hothorn MT. Package ‘ipred’. *R Package*, 2009

[70] Alfaro E, Gámez M, Garcia N (2013). adabag: an R package for classification with boosting and bagging. J Stat Softw.

[71] Andreassi C, Zimmermann C, Mitter R (2010). An NGF-responsive element targets myo-inositol monophosphatase-1 mRNA to sympathetic neuron axons. Nat Neurosci.

[72] Gumy LF, Yeo GS, Tung YC (2011). Transcriptome analysis of embryonic and adult sensory axons reveals changes in mRNA repertoire localization. RNA.

[73] Cajigas IJ, Tushev G, Will TJ (2012). The local transcriptome in the synaptic neuropil revealed by deep sequencing and high-resolution imaging. Neuron.

[74] Clegg JS, Barrios MB, Cañedo LE, Todd LE, Packer L, Jaz J (1988). The “Cytosol”: A Neglected and Poorly Understood Compartment of Eukaryotic Cells. Cell Function and Disease.

[75] Banfield RE, Hall LO, Bowyer KW, Kegelmeyer WP (2006). A comparison of decision tree ensemble creation techniques. IEEE Trans Pattern Anal Mach Intell.

