# Supplementary material for: mLoc-mRNA: predicting multiple sub-cellular localization of mRNAs using random forest algorithm coupled with feature selection via elastic net
Source: BMC Bioinformatics. 2021 Jun 24;22:342. doi: 10.1186/s12859-021-04264-8 (PMC8223360; doi:10.1186/s12859-021-04264-8)
Supplement: Supplementary file 1 — Additional file1: Table S1. Summary of the training data set, independent test set-I and II and comparison test set-I and II. [file 12859_2021_4264_MOESM1_ESM.docx]

**Additional file 1**

**Supplementary Table S1.** Summary of the training data set, independent test set-I and II and comparison test set-I and II.

| **Localization** | **Training set** | | **Independent test set-I** | | **Independent test set-II** | | **Test set-I** | | **Test set-II** | |
| --- | --- | --- | --- | --- | --- | --- | --- | --- | --- | --- |
|  | **Positive** | **Negative** | **Positive** | **Negative** | **Positive** | **Negative** | **Positive** | **Negative** | **Positive** | **Negative** |
| Cytoplasm | 1504 | 8354 | 300 | 1669 | 490 | 3974 | 86 | 139 | 464 | 619 |
| Cytosol | 1798 | 8060 | 360 | 1609 | 1037 | 3427 | - | - | - | - |
| Endoplasmic reticulum | 850 | 9008 | 170 | 1799 | 485 | 3979 | 31 | 194 | 103 | 980 |
| Exosome | 703 | 9155 | 140 | 1829 | 185 | 4279 | - | - | - | - |
| Mitochondrion | 381 | 9477 | 76 | 1893 | 12 | 4452 | 25 | 200 | 8 | 200 |
| Nucleus | 2754 | 7104 | 550 | 1419 | 1266 | 3198 | 83 | 142 | 508 | 575 |
| Pseudopodium | 180 | 9678 | 36 | 1933 | 79 | 4385 | - | - | - | - |
| Posterior | 156 | 9702 | 31 | 1938 | 121 | 4343 | - | - | - | - |
| Ribosome | 1532 | 8326 | 306 | 1663 | 789 | 3675 | - | - | - | - |

**Training set:** The dataset used to evaluate the performance of RF following five-fold cross validation. Because of the larger number of instances for the negative class as compared to its positive counterpart, there could be prediction bias towards the negative class. Thus, we employed 5 RF classifiers (instead of one) for each localization. In each RF classifier, all the instances of the positive class and an equal number of instances randomly drawn from the negative class were utilized. A majority voting strategy was applied for the final prediction i.e., if an instance was predicted to a certain class in 3 out of 5 RF classifiers the instance was said to be predicted in that class.

**Independent test set-I and II**: The datasets used to evaluate the performance of the trained RF model.

**Test set-I and II:** The datasets used to compare the performance of the proposed approach with that of mRNALoc. It contains the dataset of 4 localizations that are common to our study and mRNALoc.
